# The Power of a Soccer Ball: A Traumatic Open Finger Dislocation—A Rare Case Presentation

**DOI:** 10.1155/2015/698928

**Published:** 2015-08-16

**Authors:** Turan Cihan Dülgeroğlu, Hasan Metineren, Ekrem Aydın, Ayşegül Dülgeroğlu

**Affiliations:** ^1^Department of Orthopaedics and Traumatology, Dumlupinar University School of Medicine, 43270 Kutahya, Turkey; ^2^Department of Radiology, Evliya Çelebi Educational Research Hospital, 43050 Kutahya, Turkey

## Abstract

Proximal interphalangeal joint dislocations are injuries observed frequently and caused by axial loading on the finger in the extension. In this paper we present a traumatic open finger dislocation due to a ball hitting a wrestler. It was successfully treated with reduction and the volar plate and collateral bond fixation were applied with absorbable sutures.

## 1. Introduction

Proximal interphalangeal joint dislocations are injuries observed frequently and caused by axial loading on the finger in the extension [1]. As a result of such traumas applied on the finger which is in hyperextension, many injuries may occur like incomplete volar plate injury, collateral bond injury, extension or flexor tendon rupture, and traumatic extensor tendon dislocation. Volar plate rupture in the mid-phalanx or bone fractures with small fragments may also be observed [2]. Closed reduction, external fixation, splint, pinning, open reduction, and volar plate fixation may be applied in the treatment. The primary aim of the treatment after such injuries is the stability of the joint. If such injuries are not treated well or are neglected, they may cause pain in the finger, stiffness, degenerative arthritis, and instabilities in the future [3].

## 2. Case Report

**Figure 1:**
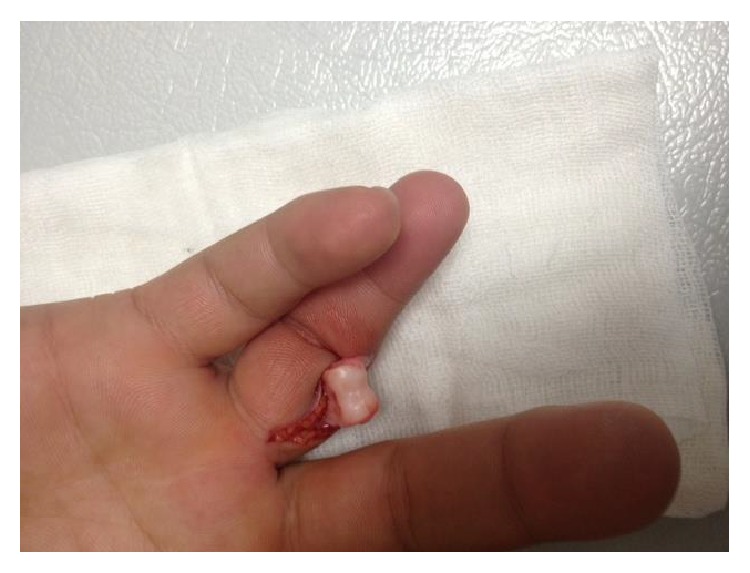
Injury showing open finger dislocation.

**Figure 2:**
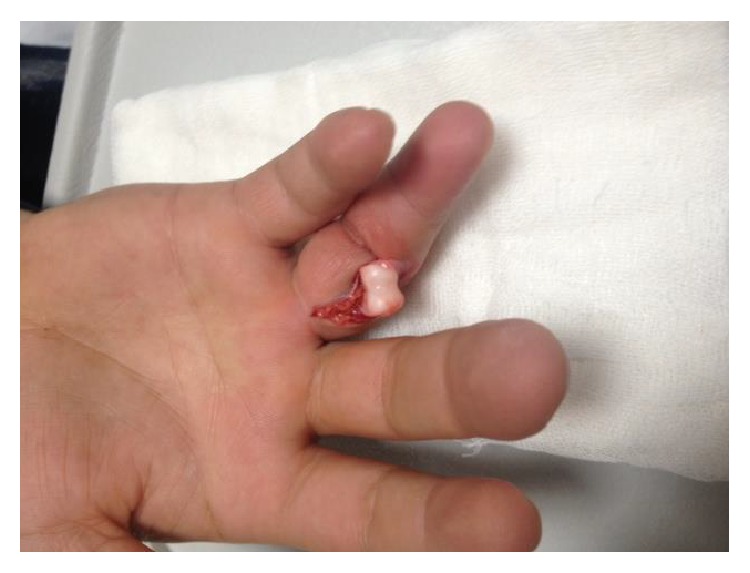
Injury showing open finger dislocation.

**Figure 3:**
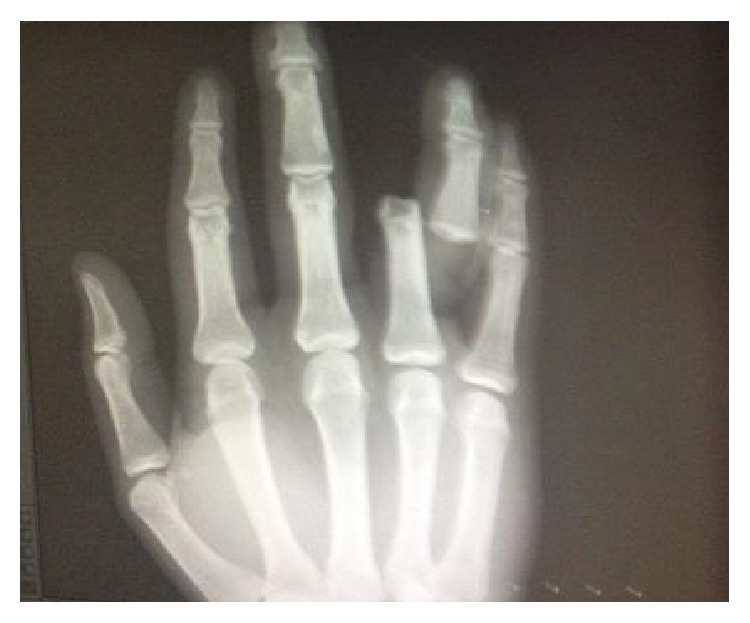
X-ray showing finger dislocation.

**Figure 4:**
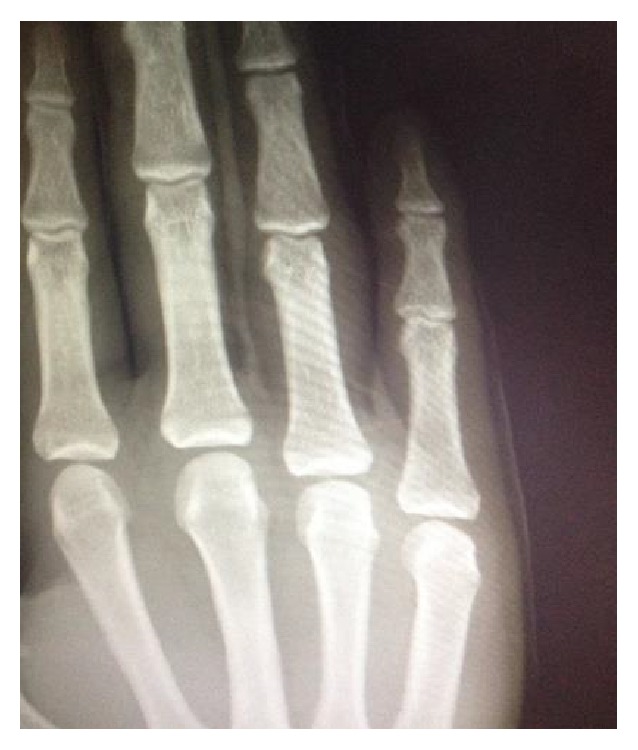
X-ray showing reduced finger.

**Figure 5:**
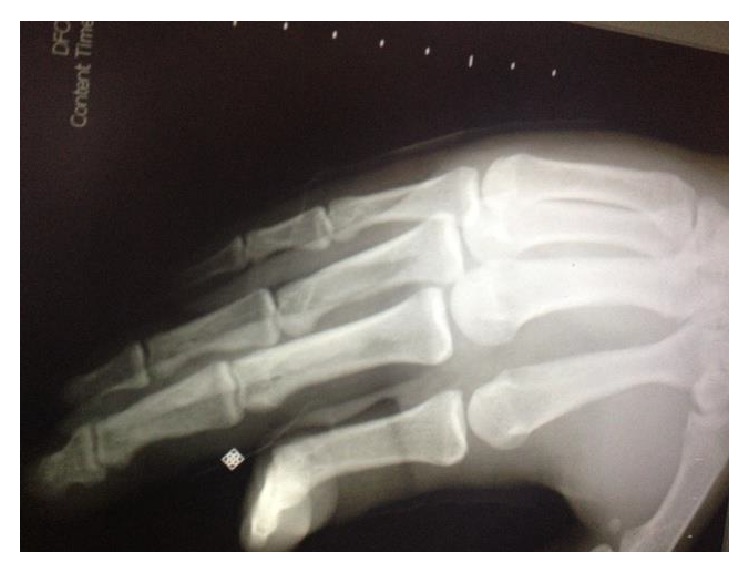
X-ray showing reduced finger.

**Figure 6:**
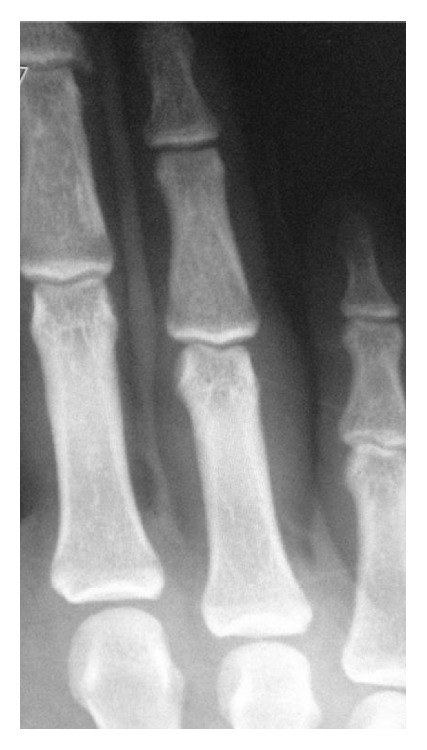
At the end of 6 months, X-ray showing joint congruity.

22-year-old male patient from Turkey, who was a wrestler, applied to the Emergency Service with joint open dislocation in finger due to a ball hitting his extension in his right hand, while he was playing football as a goalkeeper without gloves in an astro pitch. He had a proximal interphalangeal joint dislocation injury in his hand in the 4th finger in radial side stretching to the proximal phalanx and accompanied by nearly 1,5 centimeters laceration (Figures 1, 2, and 3). There was no neurovascular deficit in the finger of the patient. Washing and debridement were applied to the open joint of the patient. There was damage in the volar plate and in radial side collateral ligament. The volar plate and collateral bond fixation were applied with absorbable 5-0 PDS (PDS, Ethicon, San Angelo, TX, USA) sutures and with no need for periosteum pull-out sutures with the modified Kessler suture technique. After the reduction, the patient was kindly taken to the PIP joint extension application and the joint stability and neurovascular examinations were performed. Postoperative proximal interphalangeal joint was stable and to prevent joint from Kirschner wire traumas, Kirschner wire fixation was not implemented (Figures 4 and 5). Dorsal aluminum split was applied to the patient in postoperative 30 degrees flexion. The patient was given a postoperative antibiotherapy and wound area was examined. In the 4th week he was taken to the physical treatment program. At the end of the 6th month, the patient was able to start his active sport life fully again. Finger's flexion and extention range of motion was full degrees (Figure 6). However there was no stiffness, pain, or chronic instability. In addition, postoperative scarring was minimal.

## 3. Discussion

The main aim in PIP joint traumatic dislocations must be a concentric joint stability. Patients and doctors strive for obtaining perfect results. Many patients need rehabilitation treatment in order to reach the full ROM capacity again. Splinting in extension may be applied in these patients as a classic textbook knowledge; however, a joint staying in a long-term instable position which is not anatomical will cause contractures in the joint and may be predisposed to resistant and extra injuries in further time [4]. In PIP traumatic joint dislocations, closed reduction—fixation with K-wire, open reduction—fixation with K-wire, cerclage wire, volar plate arthroplasty, external fixation, and many other treatment methods have been defined [5]. Each treatment method has its own advantages and disadvantages within itself. In our case, since the patient had a joint open dislocation, the volar plate and collateral bond were fixed with absorbable sutures. However, if nonabsorbable sutures were used in the patient for treatment, fibrosis, granuloma, and other similar complications could be observed due to the reaction of the body to suture materials [6].

In this case report, we explained the case of an open PIP joint injury developed in the hand upon a football hit, while the patient, who dealt with wrestling, was playing football as the goalkeeper without wearing gloves. Although these types of injuries may be observed in astro pitch matches, no case reports were observed upon the literature scan talking about open PIP joint dislocation due to a ball hitting. In our opinion, the patient's playing as the goalkeeping without wearing gloves may be one of the reasons that triggered the PIP joint open dislocation. In such games, if the sportsmen start the games with proper equipment, the damage that may occur in their bodies may be decreased to a minimum level and even be avoided.
